# The Role of PoCUS in the Diagnosis and Early Monitoring of Cardiogenic Shock in the Emergency Department: A Narrative Review

**DOI:** 10.31083/RCM50103

**Published:** 2026-08-12

**Authors:** Laura Giovenali, Emanuele Guerrieri, Lorenzo Falsetti, Silvia Santini, Giulia Pierdomenico, Vincenzo Zaccone, Giovanna Viticchi, Gianluca Moroncini

**Affiliations:** ^1^Emergency Medicine Residency Program, Università Politecnica delle Marche, 60121 Ancona, Italy; ^2^Clinica Medica, Dipartimento di Scienze Cliniche e Molecolari, Università Politecnica delle Marche, 60121 Ancona, Italy; ^3^Internal and Subintensive Medicine Department, Azienda Ospedaliero-Universitaria delle Marche, 60126 Ancona, Italy; ^4^Clinica Neurologica, Dipartimento di Medicina Sperimentale e Clinica, Università Politecnica delle Marche, 60121 Ancona, Italy

**Keywords:** ultrasonography, echocardiography, cardiogenic shock, emergency department, early diagnosis, diagnosis, cardiogenic shock classification

## Abstract

Cardiogenic shock (CS) is the most serious heart failure manifestation, characterised by a significant risk of morbidity and mortality despite improving diagnosis and treatment: early recognition and accurate hemodynamic phenotypization are crucial to improve its poor prognosis. Recently, point-of-care ultrasound (PoCUS) has evolved as a key method in emergency settings for fast shock assessment. Evidence indicates that PoCUS improves the clinician’s diagnostic accuracy by identifying ventricular dysfunction, valvular disease, complications of myocardial infarction, loading conditions, and systemic congestion. Multiorgan protocols that combine cardiac, pulmonary, and vascular assessments enable differentiation among cardiogenic, obstructive, hypovolemic, and distributive shock. Echocardiographic parameters—including ventricular function, volume indices, and dynamic measures such as stroke volume and cardiac output—can guide fluid management, vasoactive therapy, and circulatory support. Limitations include operator dependence and potential imaging challenges due to patient factors. When combined with clinical and laboratory data, PoCUS is a fundamental tool for early CS assessment. With this paper, we aimed to describe the role of PoCUS in the differential diagnosis of undifferentiated shock, confirming or ruling out CS, assessing CS prognosis, guiding appropriate treatment and enabling hemodynamic monitoring. Integrating PoCUS elements with validated classifications, such as the Society for Cardiovascular Angiography and Interventions (SCAI) CS stages, can be useful for improving CS management. This review highlights validated PoCUS markers, practical PoCUS applications within the SCAI framework, and areas for further research, emphasising the increasing role of PoCUS in personalised, physiology-guided management of CS in the emergency department.

## 1. Introduction

### 1.1 Definition, Epidemiology and Socioeconomic Implications

Cardiogenic shock (CS) is the most serious manifestation of acute heart failure (AHF), with no universally accepted definition [1]. According to the current operative framework, CS can be defined as “a clinical syndrome resulting from primary cardiac dysfunction—due to impaired myocardial contractility or mechanical cardiac abnormalities—leading to reduced cardiac output and systemic tissue hypoperfusion, with a spectrum of clinical severity ranging from patients at risk to those with refractory circulatory collapse” [2]. A more recent academic definition classifies CS as “a state of circulatory failure caused by primary cardiac dysfunction, characterised by persistent hypotension or the need for pharmacologic or mechanical circulatory support to maintain adequate blood pressure, in conjunction with objective evidence of tissue hypoperfusion” [3].

CS accounts for 2–5% of AHF cases, representing 14–16% of intensive care unit (ICU) subjects [4]. In the US, 40,000–50,000 cases occur annually [5]. European data show an increasing incidence: in Germany, CS incidence rose from 33.1 to 51.7 per 100,000 person-years between 2005 and 2017 [6]. The observed increase may reflect better diagnosis and healthcare access. Higher CS rates are reported in women, Asians or Pacific Islanders, and elderly patients, over 75 years old [7].

CS mortality remains high: in-hospital death is estimated between 30 and 60% of the cases and often occurs within 24 hours of emergency department (ED) admission. An Italian ICU study reported a 44.8% in-hospital death rate [8]. Without prompt intervention, CS can progress to multiorgan failure and death. However, especially in the ED, early detection of cases at risk or during the initial stages of CS and appropriate treatment of predisposing diseases remains challenging, contributing to early mortality [9,10]. One-year mortality of survivors to the acute event reaches 50–60%, with 70–80% of subjects dying within 30–60 days from hospital discharge [11]. In the US, patients with myocardial infarction-related CS (AMI-CS) show a mortality of 40% at one month and 50% at one year [5]. Acute coronary syndromes (ACS) cause 81% of CS (ACS-CS), developing in 5–10% of subjects with ACS. Those with ST-elevated ACS have twice the risk compared to non-ST-elevated ACS [7,12]. Nonetheless, US and Canadian data indicate a decline in ACS-CS and AMI-CS and a relative increase in non-ischemic causes such as acute decompensated heart failure (ADHF), cardiomyopathies, arrhythmias, and valve disease [13].

CS is associated with high rehospitalisation rates and socioeconomic burden. Subjects developing CS after myocardial infarction (MI) have an 18.6% readmission risk within 30 days, mainly due to AHF, mechanical complications or recurrent MI. Predictors include female sex, low socioeconomic status, mechanical support use, and arrhythmias [14]. Patients with non-ischemic CS generally exhibit better in-hospital survival rates; however, they tend to have increased resource utilisation and a higher disease burden, which includes ADHF and arrhythmias [13].

### 1.2 Classification and Pathophysiological Background

CS is traditionally classified by aetiology and subdivided into three main categories [1]. In the first, CS is associated with acute cardiovascular diseases and their complications, in particular ACS-CS, AMI-CS and mechanical complications occurring in the post-MI phase. The second category is characterised by non-infarction-related CS, which is defined by the absence of ACS or AMI and is typically represented by CS associated with bradyarrhythmia, tachyarrhythmias, severe valve diseases, aortic dissection with massive regurgitation, atrial myxoma, ADHF, myocarditis, Takotsubo syndrome, septic cardiomyopathy, myocardial contusion, drug or toxin intoxication. Lastly, CS can be secondary to non-cardiac causes, as commonly observed in the last stages of other shock types, particularly obstructive shock due to cardiac tamponade and pulmonary embolism. Another classification subdivides CS into two main categories: conditions that impair myocardial contractility and mechanical disturbances. Impaired contractility is associated with both infarction-related and non-infarction-related forms. Mechanical alterations primarily involve post-MI forms (papillary muscle rupture with secondary mitral insufficiency, ventricular septal rupture, cardiac rupture with hemopericardium), aortic dissection with massive regurgitation, atrial myxoma, and severe valve diseases (aortic stenosis, mitral regurgitation, mitral stenosis). These conditions may induce the failure of the right ventricle (RV), left ventricle (LV), or both chambers, leading to a reduction in cardiac output (CO), cardiac index (CI), systemic congestion, and peripheral hypoperfusion. Hypoperfusion leads to multiorgan failure, represented by myocardial ischemia, cerebral ischemia, renal failure, liver injury, and intestinal ischemia that promotes bacterial translocation and systemic inflammatory response syndrome. LV failure increases left atrial pressure, resulting in pulmonary congestion and edema. This pathophysiological process subsequently aggravates the risk of ventilator-associated pneumonia and exacerbation of systemic inflammatory response syndrome [15]. RV failure raises right atrial and central venous pressure, impairing venous return, worsening heart failure, and damaging renal, hepatic, and intestinal function [15]. Without timely recognition and intervention, these processes can result in multiorgan failure and death.

### 1.3 Current Methods in CS Diagnosis and Treatment: The Role of SCAI Classification

In 2019, the Society for Cardiovascular Angiography and Interventions (SCAI) introduced a classification system based on uniform clinical, biochemical, and hemodynamic criteria to improve CS diagnosis, enable risk stratification, standardise clinical communication, and help clinicians manage patients at the bedside, considering clinical, biochemical and hemodynamic indicators, as shown in Table 1 [2]. The SCAI Shock Criteria were principally developed for bedside CS assessment, with stages A and B emphasising early detection of new cases, while stages C through E pertain to both acute and acute-on-chronic presentations.

**Table 1.** **2019 SCAI Criteria for diagnostic and prognostic severity classification of CS**.

SCAI stage	Description	Physical examination	Biochemical markers	Hemodynamics
A – At Risk	• Asymptomatic • At risk for CS (large acute MI, previous MI, ADHF or AHF)	• Normal JVP • Warm, well perfused • Strong distal pulses • Normal mentation • Clear lung sounds	• Lactate: normal • Renal function: baseline • Laboratory Exams: normal	• Normotensive • SBP ≥100 mmHg • CI ≥2.5 L/min/m^2^ • CVP ≤10 mmHg • PCWP ≤15 mmHg • SvO_2_ ≥65%
B – Beginning CS	• Hemodynamically unstable (relative hypotension or tachycardia) • No signs of hypoperfusion	• Elevated JVP • Warm, well-perfused • Strong distal pulses • Normal mentation • Lung rales	• Lactate: normal or mildly increased • Renal function: minimally impaired • Laboratory Exams: mild increase in NPs	• *Hypotension* SBP <90 mmHg or MAP <60 mmHg or baselines drop ≥30 mmHg • *Tachycardia* HR ≥100 bpm
C – Classic CS	• Hypoperfusion that requires treatments beyond volume resuscitation • Presence of relative hypotension is characteristic	• Volume overload • Unwell appearance • Altered mental status • Feeling of impending doom • Cold, clammy, ashen, mottled, dusky extremities, delayed capillary refill	• Lactate: ≥2 mmol/L • Renal function: 1.5× baseline or >0.3 mg/dL; GFR reduction >50% • Laboratory Exams: NPs and LFTs moderately increased	• CI <2.2 L/min/m^2^ • PCWP >15 mmHg
D – Deteriorating	• Similar to stage C but worsening despite treatment	• Worsening hypoperfusion • Initial treatment failure	• Lactate: ≥2 mmol/L, increasing • Renal function: impaired and worsening • Laboratory Exams: rising LFTs and NPs	• Perfusion is maintained by vasopressor drugs increase or MCS initiation
E – Extremis	• Actual or impending circulatory collapse	• Unconscious • Near pulselessness • Cardiac collapse • Multiple defibrillations	• Lactate: ≥8 mmol/L, severe acidosis (pH <7.2, base deficit >10 mEq/L)	• Profound hypotension despite maximal support • Need for bolus vasopressors • CPR often required

Legend: Adapted with permission from [2]. ADHF, acute decompensated heart failure; AHF, acute heart failure; CI, cardiac index; CS, cardiogenic shock; CPR, cardiopulmonary resuscitation; GFR, glomerular filtration rate; HR, heart rate; JVP, jugular vein pressure; LFT, liver function tests; MAP, mean arterial pressure; MCS, mechanical circulatory support; MI, myocardial infarction; NP, natriuretic peptides; PCWP, pulmonary capillary wedge pressure; SBP, systolic blood pressure; SvO_2_, mixed venous oxygen saturation.

The Shock Academic Research Consortium (SHARC) has recently suggested a methodological framework to harmonise CS research and enhance consistency across studies and registries, focusing on standardising the definition of CS and oriented toward providing background for future CS studies [3]. To fill the gaps present in the original SCAI classification and improve in-hospital mortality prediction for CS with real-world data, a more recent methodological refinement derived from the CS Working Group registry (SCAI-CSWG) incorporated quantitative parameters with predefined thresholds and treatment intensity. This approach suggests the use of easily accessible bedside parameters—such as systolic and mean blood pressure, lactate levels, alanine aminotransferase (ALT), pH, treatment intensity, and out-of-hospital cardiac arrest—to standardise disease stage definitions, improve prognostic stratification and guide therapeutic decisions, as shown in Table 2 [16]. The SCAI method aims to enhance clinical communication and decision-making through standardised terminology while reducing the risk of under-treatment, which is more prevalent in stages A and B of the disease.

**Table 2.** **2022 SCAI-CSWG criteria to define cardiogenic shock stages, (derived from the Cardiogenic Shock Working Group)**.

Stage	Hypotension	Hypoperfusion	Treatment intensity	Hemodynamics
SCAI A *Haemodynamically stable* SCAI-CSWG A *Haemodynamically stable*	• No hypotension	and • No hypoperfusion	and • No drugs and • No devices	• No shock • Hemodynamically stable
SCAI B *Haemodynamically unstable* SCAI-CSWG B *Hypotensive or hypoperfusing and untreated*	• 60 < SBP < 90 mmHg or • 50 < MAP < 65 mmHg	or • 2 < Lactate < 5 mmol/L or • 200 < ALT < 500 U/L	and • No drugs and • No devices	• Early shock • Hemodynamically unstable • Normotensive hypoperfusion
SCAI C *Hypoperfusion defines shock* SCAI-CSWG C *Hypotensive and hypoperfusing or treated*	• 60 < SBP < 90 mmHg or • 50 < MAP < 65 mmHg	and • 2 < Lactate < 5 mmol/L or • 200 < ALT < 500 U/L	and • No drugs and no devices or • 1 drug or 1 device *without *persistent hypotension	• Classic shock • No stabilization despite initial treatment
SCAI D *Failure to stabilise with initial therapy* SCAI-CSWG D *Failure to stabilise with initial therapy*	• 60 < SBP < 90 mmHg or • 50 < MAP < 65 mmHg	and • Lactate 5–10 mmol/L or • ALT >500 U/L	or • Total of 2–5 drugs ordevices or • 1 drug or device *with* persistent hypotension	• Worsening hemodynamics • Escalating support
SCAI E *Extremis/refractory shock* SCAI-CSWG E *Extremis/refractory shock*	• SBP <60 mmHg or • MAP <50 mmHg	or • Lactate >10 mmol/L or • pH <7.2	or • ≥3 drugs or ≥3 devices or • OHCA	• Extremis or refractory shock

Legend: Adapted with permission from [16]. ALT, alanine aminotransferase; CSWG, Cardiogenic Shock Working Group; MAP, mean arterial pressure; OHCA, out-of-hospital cardiac arrest; SBP, systolic blood pressure; SCAI, Society for Cardiovascular Angiography and Intervention.

Early identification and treatment of the underlying cause remain essential to improve CS outcomes, adopting SCAI staging to guide risk stratification and therapeutic escalation.

• Subjects in SCAI stages A and B should be assessed for the risk of developing CS and treated according to standard guidelines for their condition, with no hemodynamic support.

• Patients with SCAI stages C and D associated with ACS-CS or AMI-CS should be treated with percutaneous coronary intervention (PCI), favouring culprit-lesion-only revascularisation, with hemodynamic support with one or more inotropic or vasopressor drugs and mechanical circulatory support devices (MCS), if necessary. Regarding the timing of PCI, the SHOCK trial showed no short-term mortality benefit but significant medium- and long-term survival gains with urgent revascularisation. Regarding PCI extension, the CULPRIT-SHOCK trial demonstrated that lesion-specific PCI lowered both mortality and renal replacement therapy compared to multiple coronary PCI in patients with AMI-CS or ACS-CS [17,18].

• Patients with non-infarction-related CS with reversible causes in SCAI stages from B to E warrant targeted interventions: synchronised cardioversion for tachyarrhythmias, pacing for bradyarrhythmia, cardiac surgical repair for mechanical complications, and aetiology-specific therapies, such as fibrinolysis, antibiotics and corticosteroids, where indicated [19,20].

Within a SCAI stage-based management framework, hemodynamic treatment should be tailored to shock severity and phenotype. Hemodynamic management may be initiated with cautious fluid optimisation in the early stages when preload responsiveness is demonstrated, and congestion is absent (SCAI B–C); vasopressors and inotropes should be escalated for persistent hypotension or hypoperfusion (SCAI D–E), with de-escalation targeted. MCS is indicated for refractory D-E stages as a bridge to recovery or transplant. Organ support should also include lung-protective ventilation, with low PEEP in RV failure, and continuous venovenous ultrafiltration in acute kidney injury [10,21,22].

### 1.4 Point of Care Ultrasound: Usefulness and Applications in Cardiogenic Shock

Point-of-care ultrasound (PoCUS) represents a diagnostic method in which the clinician performs ultrasonographic assessments at the patient’s bedside, enabling immediate interpretation and incorporation of imaging findings into clinical decision-making [23]. This focused, goal-directed ultrasound technique was originally designed to identify specific signs of pathology, answering to easy, binary questions [24]. Over time, this method has been expanded to include a more comprehensive hemodynamic assessment, useful both for phenotypization and follow-up [25]. Thus, PoCUS is emerging as an essential adjunct to conventional assessment methods—such as medical history and physical examination—owing to its capacity to enhance diagnostic sensitivity and specificity, detect conditions not apparent through traditional means, but also as a valuable method to monitor hemodynamic parameters, assess therapeutic responses, and guide invasive procedures. PoCUS provides valuable, novel information in acute and potentially life-threatening situations and can influence clinical decision-making. It is particularly instrumental when the patient’s volume status is indeterminate [25,26]. PoCUS substantially advances critical care practice by precisely characterising the aetiology of shock, guiding therapeutic decision-making, monitoring patient response, and facilitating the identification of specific hemodynamic disturbances. Additionally, it enables dynamic, real-time guidance of interventions in hemodynamically unstable patients [27]. In the context of CS, PoCUS enables rapid assessment of cardiac morphology and function, identification of ventricular dysfunction, valvular abnormalities, pericardial effusion, ventricular performance and volume status. This method is useful not only for differential diagnosis of shock and early detection of underlying CS aetiologies but also for obtaining valuable information on hemodynamic parameters in specific settings, such as the ED, where advanced hemodynamic monitoring is impractical [28]. However, despite its growing integration into clinical practice, a standardised approach to PoCUS applications in real-world CS scenarios remains limited.

Aims: This narrative review aims to clarify the role of PoCUS in aiding differential diagnosis, confirming or excluding CS, guiding treatment and supporting serial hemodynamic assessments. Additionally, it explores how PoCUS-derived parameters can be integrated into current clinical staging systems, such as the SCAI classification, to enhance risk stratification and inform personalised therapeutic strategies. Integrating validated echocardiographic markers with multiorgan protocols, we propose a practical interpretive framework for bedside clinicians that bridges PoCUS findings and biochemical and hemodynamic data to enhance early phenotyping and reduce mortality in this high-risk population. This structured approach ensures methodological coherence across the manuscript, linking diagnostic accuracy, SCAI stage-specific applications, and evidence gaps for future prospective validation.

## 2. Materials and Methods

The study group searched PubMed/MEDLINE, Web of Science, and Google Scholar for case reports, reviews, meta-analyses, guidelines, and original research articles from January 1, 2015, to January 1, 2026. We used MeSH major terms and considered: “Shock”[Mesh], “Shock, Cardiogenic”[Mesh], “Shock, Septic”[Mesh], “Heart Failure”[Mesh], “Heart Failure, Diastolic”[Mesh], “Heart Failure, Systolic”[Mesh], “Acute Coronary Syndrome”[Mesh], “Pulmonary Embolism”[Mesh], “Pneumothorax”[Mesh], “Point-of-Care Systems”[Mesh], “Point-of-Care Testing”[Mesh], “Cardiac Tamponade”[Mesh], “Ultrasonography”[Mesh], “Echocardiography”[Mesh], “Cardiopulmonary Resuscitation”[Mesh], “Resuscitation”[Mesh], “Tachycardia, Ventricular”[Mesh], “Tachycardia, Supraventricular”[Mesh], “Bradycardia”[Mesh], “Heart Rupture”[Mesh], “Heart Rupture, Post-Infarction”[Mesh], “Mitral Valve Insufficiency”[Mesh], “Aortic Dissection”[Mesh], “Ultrasonography, Doppler”[Mesh], alone and in combination to obtain a list of papers. The working group included case series, original research articles, narrative reviews, randomized controlled trials, systematic reviews, and meta-analyses published within the last 15 years in the English language, focusing on adult patient populations. The panel of reviewers prioritized the inclusion of studies published during this period; however, they did not exclude highly cited older reports. Articles published prior to this timeframe, non-peer-reviewed preprints, publications in languages other than English, and studies involving patients under 18 years of age were excluded. The reference lists of articles identified through this search strategy were independently reviewed by three authors (E.G., L.G., and L.F.), with the entire working group deliberating to select the most pertinent references. Given that this constitutes a non-systematic narrative review, the selection process was conducted through a consensus approach.

## 3. PoCUS in Shock: From Rapid Diagnosis to Hemodynamic Guidance

### 3.1 Strengths and Pitfalls of PoCUS in the Differential Diagnosis of Shock

Although physical examination alone often lacks sufficient reliability to identify the aetiology of undifferentiated shock, evidence increasingly supports PoCUS as a complementary diagnostic tool in hypotensive patients presenting to the ED. Over the past few years, PoCUS has gained broad acceptance and is now widely recognised as a standard-of-care modality for the evaluation of critically ill patients, providing timely insights into the underlying pathophysiology of shock [29,30,31]. Circulatory failure is classically categorised into four main shock types: obstructive (OS), cardiogenic (CS), distributive (DS), and hypovolemic (HS), each requiring distinct therapeutic strategies. This clinical necessity has driven the integration of standardised PoCUS protocols in emergency and critical care, optimising rapid and accurate identification of shock aetiology [32,33,34]. Patients with undifferentiated shock derive the most significant benefit from such protocols [32,35,36,37], particularly those with hemodynamic instability in the ED or ICU, where timely diagnosis is directly linked to survival [31,38,39]. A recent systematic review of six studies demonstrated that PoCUS integration with clinical assessment significantly enhances diagnostic accuracy in undifferentiated shock, increasing it from 45–60% to 80–89%. Additionally, this integration was associated with increased clinician confidence [31]. In a comprehensive evaluation of PoCUS diagnostic performance, researchers analysed 30 studies involving more than 3000 patients presenting with shock. Pooled data indicate high accuracy in distinguishing among shock subtypes, with high sensitivity (77%–95%) and specificity (92%–99%). The highest diagnostic precision was observed in cases of HS, CS and OS [32,37]. PoCUS sensitivity and specificity for etiologies were, respectively, 80% and 98% for OS, 78% and 96% for CS, 90% and 92% for HS, and 79% and 96% for DS. PoCUS proved useful for ruling out shock subtypes, as negative likelihood ratios (LRs) were around 0.2. It was also effective for ruling in shock subtypes, with positive LRs exceeding 10 for most shocks, especially OS.

Limitations of PoCUS in differential diagnosis encompass operator dependence, variability in training, lack of standardised protocols, and contextual factors, highlighting the necessity of integrating PoCUS within a comprehensive clinical, multidisciplinary framework to improve diagnostic accuracy and management [32,37,40,41]. To optimise diagnostic performance in the ED, attention has shifted toward developing and validating integrated PoCUS protocols. The most widely validated point-of-care protocols for differential diagnosis of shock in the emergency department include RUSH (Rapid Ultrasound in Shock), FATE (Focus Assessed Transthoracic Echo), and SHoC (Sonography in Hypotension and Cardiac Arrest). Among these, the RUSH protocol is the most extensively studied and clinically implemented, offering a structured assessment of the heart (“pump”), inferior vena cava (IVC) and lungs (“tank”), and major vessels (“pipes”) [38,39,42]. Recent meta-analyses indicate that standardised PoCUS protocols, such as RUSH and SHoC, significantly improve diagnostic accuracy in differentiating shock types. Pooled sensitivity and specificity for PoCUS were 95% and 98% for CS, 94% and 99% for OS, 90% and 95% for HS, and 78% and 97% for DS [32,37]. The RUSH protocol appears faster and more reliable than traditional clinical assessment, particularly for OS and CS, and is therefore the most validated and recommended tool for differentiating the cause of shock in the ED [32,37,39]. The FATE protocol is primarily validated for assessing ventricular function and preload, whereas the SHoC protocol is designed explicitly for cardiac arrest and severe hypotension scenarios [38,39]. Patients with hypotension or undifferentiated shock derive the greatest diagnostic benefit from these protocols in the ED or in the ICU, where rapid diagnostic decision-making is critical for survival [39,42]. Differences among the main standardised protocols adopted for the rapid assessment of the shocked patient are summarised in Table 3. These three protocols are specific for the non-traumatised patient, whereas the extended Focused Assessment with Sonography in Trauma (eFAST) protocol, which is more HS-oriented, should be selected to evaluate shocked trauma patients admitted to the ED.

**Table 3.** **Different standardised PoCUS protocols to study undifferentiated shock**.

Protocol	Key features	Applications	Findings
RUSH	Multiorgan assessment: • the “pump” (heart) • the “tank” (volume, lungs, IVC) • the “pipes” (aorta, large vessels, particularly leg veins) Most comprehensive evaluation for undifferentiated shock	• Characterization of hypovolemic, cardiogenic, obstructive and distributive shock • Emergency care • Cardiac arrest	• Ventricular dysfunction • Tamponade • IVC dilation • Pleural or abdominal effusions • Aortic aneurysm and dissection • Pulmonary embolism • Pneumothorax
FATE	Focused echocardiography: • performed in 4 standard views • cardiac function-oriented • less extensive than RUSH	Rapid cardiac assessment for: • CS • Acute heart failure • ICU monitoring	• Ventricular function • Pericardial effusion • Valvular morphology • Tamponade • Volume estimation
SHoC	Resuscitation-oriented protocol for hypotension and cardiac arrest, integrating the assessment of: • heart • lung • abdomen • vessels	• Undifferentiated shock • Cardiac arrest • Severe hypotension • Emergency care	• Cardiac function • Tamponade • Pulmonary embolism • Pneumothorax • Abdominal effusion • Volume status

Legend: CS, cardiogenic shock; ICU, intensive care unit; IVC, inferior vena cava; RUSH, Rapid Ultrasound in Shock; FATE, Focus Assessed Transthoracic Echo; SHoC, Sonography in Hypotension and Cardiac Arrest.

PoCUS can help physicians differentiate shock types. Each type of shock has its typical ultrasound characteristics, which must be interpreted considering clinical and laboratory findings in order to maximise the diagnostic accuracy:

• Cardiogenic Shock: Differentiating CS from other shock phenotypes using PoCUS depends on its capability of identifying specific cardio-thoracic findings. CS is clearly defined from the C stage of SCAI classification, considering clinical, laboratory, and hemodynamic parameters [2,43]. However, because early recognition and stabilisation are critical for reducing morbidity and mortality, it is essential to recognise CS at earlier stages of SCAI, before the onset of hypotension or end-organ hypoperfusion [44]. Guidelines and expert opinions advocate for the prompt cardiac PoCUS use to confirm CS and to identify reversible aetiologies, including cardiac tamponade or mechanical causes, such as acute valvular dysfunction [45]. ACS-CS, AMI-CS, post-MI-CS and their associated complications account for approximately 70% of cases: among these, acute or acute-on-chronic left heart failure (79%), acute mitral insufficiency (7%), ventricular septal perforation (4%), isolated RV infarction (3%), and cardiac tamponade due to free wall rupture (2%) should always be assessed. Non-ischemic etiologies are less frequently encountered and include LVOT obstruction, RV failure, myocarditis, septic myocardial depression, various cardiomyopathies, myocardial contusion, acute aortic insufficiency, and iatrogenic causes [46]. Characteristic cardiothoracic PoCUS findings include LV systolic dysfunction, regional wall motion abnormalities, reduced LVEF, ventricular dilation, pulmonary congestion (B-lines), dilated and poorly collapsible IVC, acute valvular lesions, RV dysfunction, cardiac tamponade, and stroke volume (SV) and cardiac output (CO) reduction, even with preserved LVEF [46,47]. PoCUS diagnostic accuracy may be limited in patients with mixed shock and suboptimal acoustic windows, which are common in obesity, chronic obstructive lung disease (COPD), and mechanically ventilated subjects [33,37,46,48]. PoCUS can be difficult to interpret, particularly in early CS and chronic cardiac disease, where B-MODE results should be combined with the hemodynamic profile to provide broader phenotyping [49].

• Obstructive Shock: PoCUS can rapidly identify features indicative of an acute mechanical obstruction that impairs regular cardiac filling or output. In cases of high-risk pulmonary embolism (PE), typical findings include indirect signs such as RV dilation and dysfunction, often accompanied by specific indirect signs, like 60/60 or McConnell’s sign [50]. Direct signs may also be observed: in particular, thrombi can be visualised with PoCUS within the right heart chambers or in the proximal leg veins. A large pericardial effusion accompanied by diastolic RV collapse indicates the likely presence of cardiac tamponade. Absent pleural sliding with a *lung point* is characteristic of tension pneumothorax. In the suspicion of acute aortic disease, the integration of clinical (ADD-RS score), laboratory (D-dimer), and PoCUS findings can allow for the safe exclusion of a dissection. On the other hand, visualisation of direct signs of dissection, such as a massive aortic insufficiency, an intimal flap or pathological aortic dilation can be suggestive of acute aortic disease and induce the ED physician to perform an urgent computed tomography (CT) scan. These sonographic findings are helpful in differentiating OS from other shock types with high diagnostic accuracy [32,37]. PoCUS accuracy in OS is affected by image quality, operator expertise, and patient-related factors such as obesity, COPD, mechanical ventilation, or thoracic deformities, which may impair visualisation of key findings like RV dilation or diastolic collapse [33,51]. False positives can arise from chronic conditions mimicking acute pathology (e.g., pulmonary hypertension and congenital heart diseases), pericardial effusion without tamponade, absent pleural sliding due to adhesions or fibrosis, or misinterpretation of aortic intimal flaps [23,32,37,52,53]. Integrating PoCUS findings with clinical and laboratory assessments and complementary diagnostic tests is essential to minimise misdiagnosis and guide appropriate management.

• Hypovolemic Shock: Typical findings include small, hyperdynamic cardiac chambers, a small LV with a normal LVEF, and a narrow, highly collapsible IVC, often <1 cm in diameter, with >50% inspiratory collapse, reflecting low intravascular volume. Lung ultrasound (LUS) generally shows the absence of diffuse B-lines, distinguishing hypovolemic from cardiogenic or distributive shock, while cardio-thoraco-abdominal eFAST (in trauma) or RUSH (in medical patients) assessment may detect free thoracic or abdominal fluid in cases of HS [26,54,55,56,57]. PoCUS demonstrates high diagnostic accuracy, with sensitivity around 90% and specificity between 92% and 95% [32,37]. Accuracy may be reduced in mechanically ventilated patients, in whom positive-pressure ventilation alters IVC dynamics, in subjects with obesity, COPD, or thoracic anatomical variations, and in paediatric or neonatal populations, due to physiological variability and rapid hemodynamic compensation. Classic sonographic signs may also be absent in mild or compensated hypovolemia, and intra-abdominal conditions such as ascites or elevated intra-abdominal pressure can further distort IVC morphology and collapsibility [58,59,60,61,62]. These considerations highlight the importance of integrating PoCUS findings with the clinical context and complementary diagnostics to guide accurate and timely management in critically ill patients.

• Distributive Shock: DS is caused by a peripheral vascular resistance drop, often complicated by concurrent myocardial dysfunction and hypovolemia, which challenges comprehensive hemodynamic assessment [63]. Consequently, PoCUS protocols demonstrate low accuracy and interobserver agreement to diagnose DS [32,37,63,64]. Ultrasonographic evaluation typically does not identify a reduced SV, which is typically normal or slightly elevated; the CI is commonly greater than 2.2 L/min/m^2^ [26]. PoCUS can aid in diagnosing distributive shock by identifying preserved or hyperdynamic LV function, normal or enlarged cardiac chambers, and a frequently narrow, highly collapsible IVC. In the early stages of DS, CO is often elevated due to the reduction of systemic vascular resistance. Septic myocardial dysfunction with mixed-type shock (CS and DS) may occur in advanced stages, with preserved or increased systolic function in the early phases. Unlike other shock types, DS lacks mechanical obstruction such as tamponade, pulmonary embolism, or tension pneumothorax. It does not present with dilated chambers and depressed systolic function as in CS, nor the small, hyperdynamic chambers and collapsible IVC typical of HS. However, IVC collapsibility may occasionally be observed. Pulmonary congestion is typically absent unless fluid overload is present. Thus, PoCUS diagnosis relies on excluding features of other shock types while recognising preserved or hyperdynamic cardiac function, often accompanied by signs of peripheral vasodilation and relative hypoperfusion [32,37,47,65].

Table 4 summarises the most common PoCUS findings in each type of shock, the recommended PoCUS protocol, and the obtained sensitivity and specificity. PoCUS shows a good sensitivity and specificity in each shock subtype, and can be used to guide treatment, assessing also the response to the specific therapy. The validated protocols can be applied according to the patient’s clinical context.

**Table 4.** **Different PoCUS findings according to shock subtype**.

Shock type	PoCUS findings	PoCUS protocol	Se (%)	Sp (%)
Cardiogenic	• LV or RV systolic dysfunction • Dilated cardiac chambers • Pulmonary congestion (B-lines) • Dilated and poorly collapsible IVC, integrated with VExUS score • Possible acute valvular lesions	For undifferentiated shock or acute heart failure: • RUSH • FATE	78–95	96–98
Obstructive	• RV dilation or dysfunction • Pericardial effusion with tamponade (diastolic collapse) • Absent pleural sliding (pneumothorax) • Aortic aneurysm or dissection	For undifferentiated shock, cardiac arrest, trauma: • RUSH • SHoC • eFAST	80–94	98–99
Hypovolemic	• Ventricles: small, hyperdynamic • IVC: narrow and highly collapsible • Pulmonary congestion absent • Presence of free abdominal or thoracic fluid	For undifferentiated shock, trauma, hemorrhagic shock: • RUSH • FATE • eFAST	90	92–95
Distributive	• Ventricles: normal, hyperdynamic • IVC: often small, collapsible • No pulmonary congestion (B-lines) • Peripheral vasodilation pattern	For septic shock, undifferentiated shock: • RUSH • SHoC	78–79	96–97

Legend: IVC, inferior vena cava; LV, left ventricle; RV, right ventricle; VExUS, venous excess ultrasound; RUSH, Rapid Ultrasound in Shock; FATE, Focus Assessed Transthoracic Echo; SHoC, Sonography in Hypotension and Cardiac Arrest; eFAST, extended Focused Assessment with Sonography in Trauma; Se, sensitivity; Sp, specificity.

### 3.2 Strengths and Pitfalls of PoCUS in the Diagnosis of Cardiogenic Shock

Multiple studies, including systematic reviews and meta-analyses, have substantiated the PoCUS accuracy in CS diagnosis, highlighting its significant clinical utility [29,32,66,67,68]. In a recent ED study assessing patients with shock, researchers found high concordance between PoCUS-based diagnoses and final confirmed CS diagnosis, with high sensitivity and negative predictive value [64]. Cardiac PoCUS revealed reduced LVEF in all but one patient, whose CS was due to acute severe mitral regurgitation secondary to papillary muscle rupture following acute MI. Additional CS aetiologies included severe mitral stenosis and combined aortic stenosis with regurgitation. These results highlight both the diagnostic precision and the capacity of PoCUS to detect complex valvular or mechanical complications. Several authors agree that PoCUS can aid the diagnosis, monitoring, and management of critically ill patients with CS or OS [69].

Moreover, integrating PoCUS into early diagnostic workflows markedly improves accuracy, enabling precise identification of the pathophysiological mechanisms underlying hemodynamic instability [29,31,32]. Several studies underline high PoCUS sensitivity and specificity across all major shock types and, when applied through structured multisite protocols, provide a comprehensive hemodynamic assessment that enhances bedside decision-making in critically ill patients [32,69].

Although CS accounts for only 2–5% of shock cases, mortality ranges from 30% to 60%, underscoring the importance of early aetiological recognition and timely initiation of targeted pharmacological therapy, MCS or cardiac surgery [29,31]. A recent meta-analysis reported pooled sensitivity and specificity of 86.1% and 95.8% for CS [29]. A multiorgan PoCUS improves diagnostic performance by combining cardiac, lung, and vascular imaging, thereby increasing sensitivity and specificity for identifying CS compared with isolated cardiac ultrasound [32]. PoCUS findings associated with CS include reduced LVEF, regional wall motion abnormalities, ventricular dilation, IVC dilation, pericardial effusion, and B-lines on lung ultrasound (LUS) [64,70,71]. Real-world data demonstrated high concordance between PoCUS-guided diagnoses and final discharge diagnoses in patients presenting with acute dyspnoea, suspected acute decompensated heart failure, acute coronary syndrome, or shock [72].

On the other hand, PoCUS has several limitations in the setting of CS diagnosis. Distinguishing acute from chronic cardiac dysfunction remains challenging, and false positives may occur in patients with prior MI, CHF or cardiomyopathies. In contrast, false negatives can result from a poor acoustic window or subtle myocardial abnormalities. PoCUS findings are context-dependent and must be interpreted in light of comprehensive clinical and laboratory data [66]. Another limitation concerns operator expertise, which is essential for optimising PoCUS accuracy. Targeted training reduces misclassification in LVEF assessment, and brief instructional sessions can improve recognition of regional wall motion abnormalities, underscoring the importance of structured educational programs for clinicians [73,74].

### 3.3 Strengths and Pitfalls of PoCUS in Hemodynamic Assessment of Cardiogenic Shock

CS management should be individualised according to the predominant cardiac dysfunction and guided by comprehensive hemodynamic monitoring to maintain adequate tissue perfusion while correcting the underlying cause [45,75]. Despite a substantial paucity of data in CS, hemodynamic assessment should aim to maintain mean arterial pressure (MAP) at 65 mmHg to ensure adequate tissue perfusion [76,77]. MAP can be described as the product of SV, heart rate (HR) and systemic vascular resistance (SVR) plus central venous pressure (CVP). SV is influenced primarily by three key components: preload, ventricular contractility, and afterload. CO, calculated as the product of SV and HR, is the only variable in the MAP formula that is directly associated with the tissue delivery of oxygen (DO_2_). SV and HR should be evaluated, adjusted and monitored from the first CS assessment to improve tissue perfusion and DO_2_. The other variables, SVR and CVP, albeit not directly correlated with DO_2_, should also be evaluated to further optimise volume status and reach the MAP target to optimise tissue perfusion. PoCUS allows early CS recognition, accurate hemodynamic phenotyping, and prompt shock classification, thus significantly improving outcomes [45,75,78]. An extensive list of PoCUS hemodynamic parameters is shown in Table 5 (Ref. [5,26,34,45,49,58,75,79,80,81,82,83,84,85,86,87,88,89,90,91,92,93,94,95,96,97,98,99,100,101,102,103,104,105,106,107,108,109,110,111,112,113,114,115,116,117,118,119,120,121,122,123,124,125,126,127,128,129,130,131,132,133,134,135,136,137,138,139,140,141,142,143,144,145,146,147,148,149,150,151,152,153,154,155,156,157,158,159,160,161,162,163]). However, a brief hemodynamic phenotypization can be achieved using PoCUS by assessing preload, contractility, LV performance indices, afterload, and CVP:

**Table 5.** **Main cardiac PoCUS findings and PoCUS hemodynamic assessment in CS**.

Findings	Indications and limitations
RV preload	• Indications: RV preload is crucial for guiding fluid therapy, and can be assessed with: ○ IVC size and collapsibility ○ VExUS score, that integrates Doppler data from multiple venous territories, enhancing correlation with RAP and systemic congestion to gather additional information in cases of volume overload, especially in patients with suspected significant systemic venous congestion [79,80,81,82,83,84]. • Limits: IVC diameter and collapsibility have a modest correlation with RAP, affected by technical, physiological, and patient-related factors such as ventilation, obesity, and intra-abdominal pressure [58,85,86,87,88]. VExUS requires advanced expertise, is affected by organ dysfunction, arrhythmias, and right heart disease, meaning use is limited in unstable patients [81,83,84,89]. Both assessments should be integrated with echocardiographic and clinical data: their prognostic and therapeutic role in CS remains incompletely defined [80,89,90,91].
RV contractility	• Indications: RV contractility maintains right-sided CO, adequate pulmonary perfusion and LV preload. ○ Functional parameters: TAPSE, TDI lateral tricuspid annular systolic velocity are simple, reproducible markers of longitudinal systolic function [88,92]. FAC provides a sensitive and prognostic measure of RV dysfunction [88,92,93], LVEI can help differentiate pressure from volume overload. The TAPSE/PASP ratio reflects RV–pulmonary artery coupling [92]. A multiparametric approach is recommended to optimise diagnostic accuracy in critically ill patients [88,94,95]. ○ Morphological features: RV dilation, hypertrophy, right atrial enlargement, and paradoxical septal motion support functional assessment [92,96]. • Limits: TAPSE and S’ assess only longitudinal function and are highly dependent on angle, quality, and load conditions, potentially yielding false-normal results in septal hyperkinesia, tethering or valve dysfunction [88,92,97,98]. FAC and LVEI are influenced by complex RV geometry and may not capture radial or outflow tract function. TAPSE/PASP ratio is prone to technical and inter-observer variability, particularly in CS [88,92,97].
RV afterload	• Indications: RV afterload is determined mainly by PVR, a key determinant in CS and AHF [99]. Pulmonary pressures guide fluid management and reflect left-sided congestion or decongestion. ○ Pulmonary pressure parameters: PASP provides a non-invasive estimate of RV afterload in CS, indicating increased afterload, frequently linked to RV dysfunction and worse outcomes [92,93]. A TAPSE/PASP ratio <0.4 mm/mmHg predicts CS adverse events, outperforming TAPSE or PASP alone in risk stratification by detecting early RV contractile inefficiency [100,101,102,103,104,105]. LVEI quantifies leftward septal displacement, reflecting elevated RV afterload, useful when Doppler pressures are unreliable [92,106]. • Limits: PASP estimation is limited by poor Doppler signal, RAP is influenced by technical and physiologic variability. TAPSE/PASP ratio is sensitive to load conditions, arrhythmias, mechanical ventilation, and acoustic window quality. LVEI is also limited by window quality, operator variability, and septal alterations unrelated to RV overload, such as LV pathology or constrictive pericarditis, providing only an instantaneous assessment that must be integrated with other clinical and echocardiographic data [92,106].
LV preload	• Indications: PoCUS allows a fast assessment in early shock to identify pulmonary congestion, estimate LV filling pressures, fluid responsiveness, and guide therapy [34,107,108,109,110,111,112]. ○ Increased LV preload: transmitral PW Doppler and IVC evaluation [108,113,114,115,116]; E/e′ ratio is the most validated parameter, >15 indicates a significant LVFP increase [113,117]. Cardiogenic B-lines are associated with increased LVFP. ○ Reduced LV preload: a reduced LV end-diastolic area, a preserved or hyperdynamic systolic function, low transmitral E-wave velocity, low E/e′ ratio, and a small IVC are suggestive of low preload: a passive leg raising test is often necessary to confirm a low-preload state, given the SV increase after stressed volume administration. • Limits: B-lines have reduced specificity by non-cardiogenic pulmonary pathology, while sensitivity is limited in obesity, emphysema, or alterations of thoracic anatomy [109,110,112,118]. B-lines and IVC may be normal in case of CS with hypoperfusion, in early-stage CS, in low–filling pressure phenotypes, in isolated RV failure, or in LV dysfunction without pulmonary congestion [5,26,45,112,116,119]. E/A is limited by tachycardia, atrial fibrillation, or severe valvular abnormalities. E/e′ is less reliable in severe systolic dysfunction, valvular heart disease, or in patients with ventricular assist devices.
LV ejection fraction	• Indications: LVEF is fundamental in assessing systolic function in CS. ○ LVEF estimates: In CS, visual estimation (“eyeball”) and biplane Simpson method are suggested [58,75,120]. Eyeball strongly correlates with the Simpson method and shows superior reproducibility in unstable patients [121]. ○ Linear measures: When LVEF assessment is suboptimal, surrogate indices as MAPSE and S’ on lateral mitral annulus provide complementary insights [122,123,124,125]. S’ is useful in critically ill patients and demonstrates robust intra- and inter-operator reproducibility [123,126]: S’ values <7 cm/s reliably detect LVEF <45%, (Se: 93%; Sp: 87%). • Limits: LVEF evaluation is limited by acoustic window quality; MAPSE is load-dependent, less sensitive with regional wall motion abnormalities, mitral valve disease, or cardiac pacing, and requires age- and sex-specific interpretation [125,127,128]. MAPSE could remain preserved despite reduced LVEF due to compensatory circumferential contraction [123]. S’ is similarly influenced by loading conditions, regional dysfunction, valvular disease, and cardiac rhythm limiting its reliability as an isolated indicator of global systolic performance [122,129]. S’ is less affected by poor acoustic windows, but correlation with volumetric LVEF remains moderate [121,126].
LV performance	• Indications: LVOT_VTI_, SV, and CO are critical for diagnosis, prognosis and therapeutic guidance in CS. ○ LVOT_VTI_: correlates with SV, with values <14 cm suggesting SV depression, <12 cm predicting poor outcomes. In the context of low-preload states, LVOT_VTI_ variation induced by a passive leg-raising test can be used to assess fluid responsiveness. During amine or fluid treatment, LVOT_VTI_ can be used to monitor hemodynamic response [130]. ○ SV, CO and CI: are key determinants of organ perfusion, specifically, a cardiac index (CI) ≤1.8 L/min/m^2^ or a CO <3.5 L/min within the first 24 hours of shock has been linked to poor prognosis and a higher 30-day mortality risk [131,132,133]. LV performance estimate is necessary for risk stratification, therapy titration, and response monitoring [45]. Integrating performance markers enables physiologically guided, individualized management and offers superior prognostic value compared with LVEF alone [45,130,131]. • Limits: reliability depends on acoustic window quality and Doppler alignment; arrhythmias, mechanical ventilation, or valvular disease can further compromise accuracy [49,130,134]. Minor errors in LVOT diameter can propagate, producing >30% deviation relative to invasive reference methods [135,136]. Intra- and inter-operator variability are substantial, especially in severe shock or hemodynamic instability [137,138].
LV diastolic function	• Indications: LV diastolic function guides differential diagnosis of acute dyspnoea, risk stratification, therapy, and monitoring of treatment response. ○ Diastolic dysfunction markers: E/A, E/e′, pulmonary vein flow analysis, E-wave deceleration time, LAVI, and TR jet velocity are necessary for a multiparametric assessment of diastolic function [88,113,114,139,140,141]. E/A ratio identifies impaired relaxation or restrictive filling [113,142]. E/e′ ratio is a validated surrogate for LVFP [113]. Restrictive patterns and elevated LVFP are associated with poorer outcomes and lower tolerance to therapeutic interventions in CS [88,142]. Intermediate or inconclusive values require integration with other parameters, such as TRV and LAVI [114,143,144,145,146,147]. • Limits: E/A ratio is frequently compromised in the presence of tachycardia, atrial fibrillation, or EA waves fusion, conditions common in CS. E/e′ is less correlated with LVFP in severe systolic dysfunction, mitral valve disease, ventricular assist devices, or regional annular abnormalities [117,143,148]. Pulmonary venous flow is technically challenging, influenced by operator variability, hemodynamic load, atrial function, and respiration, and is less specific in atrial fibrillation, pacing, mitral regurgitation, or structural abnormalities [149,150,151,152,153]. DT and LAVI are load- and operator-dependent, unreliable in tachycardia, arrhythmias, or valvular/structural disease; DT may be confounded by EA fusion, while LAVI does not capture acute pressure changes [88,142]. TRV requires adequate regurgitation and high-quality windows, it may overestimate pressures in pulmonary disease or RV dysfunction, it correlates moderately with LVEF and can be significantly influenced by extracardiac factors and changes in hemodynamic load [114,145,146].
LV afterload LVOT obstruction	• Indications: The differential diagnosis of increased afterload includes arterial hypertension, increased aortic stiffness, aortic stenosis, and LVOTO. PoCUS allows the assessment of aortic stenosis and LVOTO, helping in differentiating dynamic from fixed forms. ○ Dynamic LVOTO: commonly associated with mitral SAM, hypovolemia, hypercontractility, reduced afterload, tachycardia, or inotropic support, is characterized by a variable gradient that is accentuated by provocative manoeuvres such as Valsalva, orthostasis, or exercise, and exhibits the classic dagger-shaped Doppler profile that can be sampled in the LVOT [154,155,156,157]. ○ Fixed LVOTO: resulting from anatomical subaortic stenosis, fibrosis, or structural abnormalities, presents a persistent prevalvular gradient that remains largely unaffected by hemodynamic changes [154,155]. Distinguishing between the dynamic and fixed forms is crucial for the therapeutic management of the patient [154,155,156,157,158]. • Limits: In CS, low SV can produce deceptively low gradients even in severe aortic stenosis, Doppler misalignment with the jet, confusion with mitral regurgitation, operator variability, patient instability, and mechanical ventilation [159]. LVOTO assessment is particularly difficult due to hemodynamic dependence and limited feasibility of provocative maneuvers; gradients are highly dynamic and may be underestimated in low preload, reduced afterload, or hypovolemia [154,155,160,161]. Distinguishing dynamic from fixed LVOTO requires high-quality imaging and Doppler artifacts may further compromise measurement [162,163].

Legend: CO, cardiac output; CI, cardiac index; CS, cardiogenic shock; DT, decay time or deceleration time; FAC, fractional area change; LV, left ventricle; LAVI, left atrial volume index; LVEF, left ventricular ejection fraction; LVFP, left ventricular filling pressure; LVOT, left ventricle outflow tract; LVOTO, left ventricle outflow tract obstruction; LVOT_VTI_, velocity-time integral at the left ventricle outflow tract; LUS, lung ultrasound; MAPSE, mitral annular plane systolic excursion; PASP, pulmonary artery systolic pressure (estimated); PVR, pulmonary vascular resistance; SV, stroke volume; RAP, right atrial pressure; LVEI, left ventricle eccentricity index; SAM, systolic anterior motion of the mitral valve; TAPSE, tricuspid annular plane systolic excursion; TR, tricuspid regurgitation; TRV, tricuspid regurgitant velocity; VExUS, venous excess ultrasound; VTI, velocity-time integral.

• Preload assessment: fluid management remains a controversial topic in CS, but preload optimisation is essential in all shock states. However, the attending physician should evaluate three specific dimensions of the shocked patient before fluid resuscitation to avoid a useless or harmful treatment. First, fluid responsiveness should always be evaluated using the passive leg raising (PLR) test, with positive results defined as ≥10% SV increase after PLR [164]. Only patients who are expected to experience a significant increase in SV after preload optimisation should be considered for fluid therapy. In this group, it is also necessary to assess fluid tolerance, which reflects the risk of developing overt fluid overload after effective fluid resuscitation. Although a theoretical construct, fluid tolerance should be assessed by PoCUS, including evaluation of subclinical congestion, B-lines on lung ultrasound, the E/e′ ratio, the dimensions and collapsibility of the IVC, and the grade of venous excess ultrasound (VExUS). The third concept is fluid overload, which is an overt lung or interstitial oedema that can be evaluated by PoCUS by assessing pleural and peritoneal free fluid, B-lines on lung ultrasound, left and right atrial size, E/e′ ratio, the dimensions and collapsibility of the IVC, and the grade of the VExUS score. Albeit controversial, preload optimization with fluids can be beneficial in CS, especially in certain subtypes as in inferior ACS-CS, RV dysfunction, and mixed-type CS, especially in the context of septic cardiomyopathy and Takotsubo syndrome [165]. Fluid responsiveness, fluid tolerance, and fluid overload should be considered variable over time and reassessed after fluid treatment or before administering further fluid boluses. Patients with CS are often fluid-overloaded, and the same parameters can be used to optimise volume status and preload in fluid-expanded states.

• Contractility assessment: global RV and LV contractility should always be assessed. LV function can be assessed using the eyeballing method and linear methods, such as mitral annular plane systolic excursion (MAPSE), as well as the volumetric Simpson technique. RV function can be assessed with tricuspid annular plane systolic excursion (TAPSE). This information should be integrated with ventricular performance metrics, such as SV, to distinguish chronic alterations that do not affect hemodynamics from acute alterations associated with a reduction in SV and CO [49].

• Afterload assessment: both RV and LV afterload should also be optimised to improve SV. An increased RV afterload can be easily synthesised by tricuspid regurgitant velocity (TRV) and its direct derivative, the pulmonary artery systolic pressure (PASP). An increased LV afterload can be assessed in left ventricular outflow tract (LVOT) obstruction, aortic stenosis, and hypertensive crisis, and is often associated with reduced LV contractility. A reduced LV afterload can be observed in CS in acute mitral insufficiency or in mixed forms of shock and is associated with low telesystolic volume and a hyperkinetic ventricle.

• Ventricular performance metrics: SV can be assessed with PoCUS by obtaining the LVOT area and multiplying it by the velocity-time integral at the LVOT (LVOT_VTI_). CO is the product of SV by HR. Optimisation of preload with fluids, contractility with inotropes, and afterload with vasopressors directly affects SV and CO. At the same time, HR regularisation can improve CO. Thus, SV and CO should always be measured as diagnostic metrics for CS but are also required to monitor treatment response.

All CS guidelines underscore the critical role of serial assessments to detect indicators of hypoperfusion, including mottling, extremity coolness, dizziness, and mental status alterations. Nevertheless, clinical assessment alone cannot reliably quantify key parameters such as SV and CO, which are central to guiding treatment [45,75,166]. PoCUS can be useful for hemodynamic phenotyping and pivotal in personalising interventions to improve MAP, thereby minimising the risks of under- or over-resuscitation. PoCUS is a rapid bedside method with high accuracy for identifying the cause of shock, including CS, and is especially useful for early hemodynamic phenotypization [32,37,53]. Furthermore, by enabling the immediate assessment of heart morphology and function, intravascular volume status, and major valvular abnormalities, PoCUS supports prompt, informed diagnostic and treatment decisions that can directly affect patient outcomes [23,167,168]. Early implementation of cardiac PoCUS is currently suggested to improve diagnostic and hemodynamic evaluation in CS [32,43,51,169,170]. In patients who remain refractory to initial resuscitative measures, serial echocardiographic assessments are useful for hemodynamic monitoring and for the early detection of mechanical complications associated with acute MI, including free wall rupture, interventricular septal defects, papillary muscle or mitral chordae rupture, and complications related to mechanical circulatory support. PoCUS serves both diagnostic and prognostic roles, bridging the gap between bedside clinical evaluation and more complex hemodynamic monitoring, with results comparable to pulmonary artery catheter-derived data [171,172].

There is a paucity of robust evidence regarding the role of PoCUS in optimising treatment across the CS spectrum, including pre-CS, compensated CS with preserved systolic blood pressure, and decompensated shock with hypotension responsive to fluid administration or inotropes. Some studies report no significant differences in the administration of inotropes or fluid therapy between pre- and post-PoCUS management [173,174]. In contrast, others highlight the critical PoCUS role in guiding therapy for patients with septic shock or undifferentiated shock [175,176,177]. These considerations underscore the need for further research to validate PoCUS-based algorithms that can not only identify shock aetiology but also stratify patients by hemodynamic severity and guide tailored interventions.

In Table 5, we present the principal PoCUS techniques that enable clinicians to perform early hemodynamic phenotypization in patients with shock, thereby guiding treatment, providing robust prognostic stratification and monitoring treatment response.

### 3.4 The Usefulness of Integrating PoCUS in SCAI Classification

**Fig. 1.**
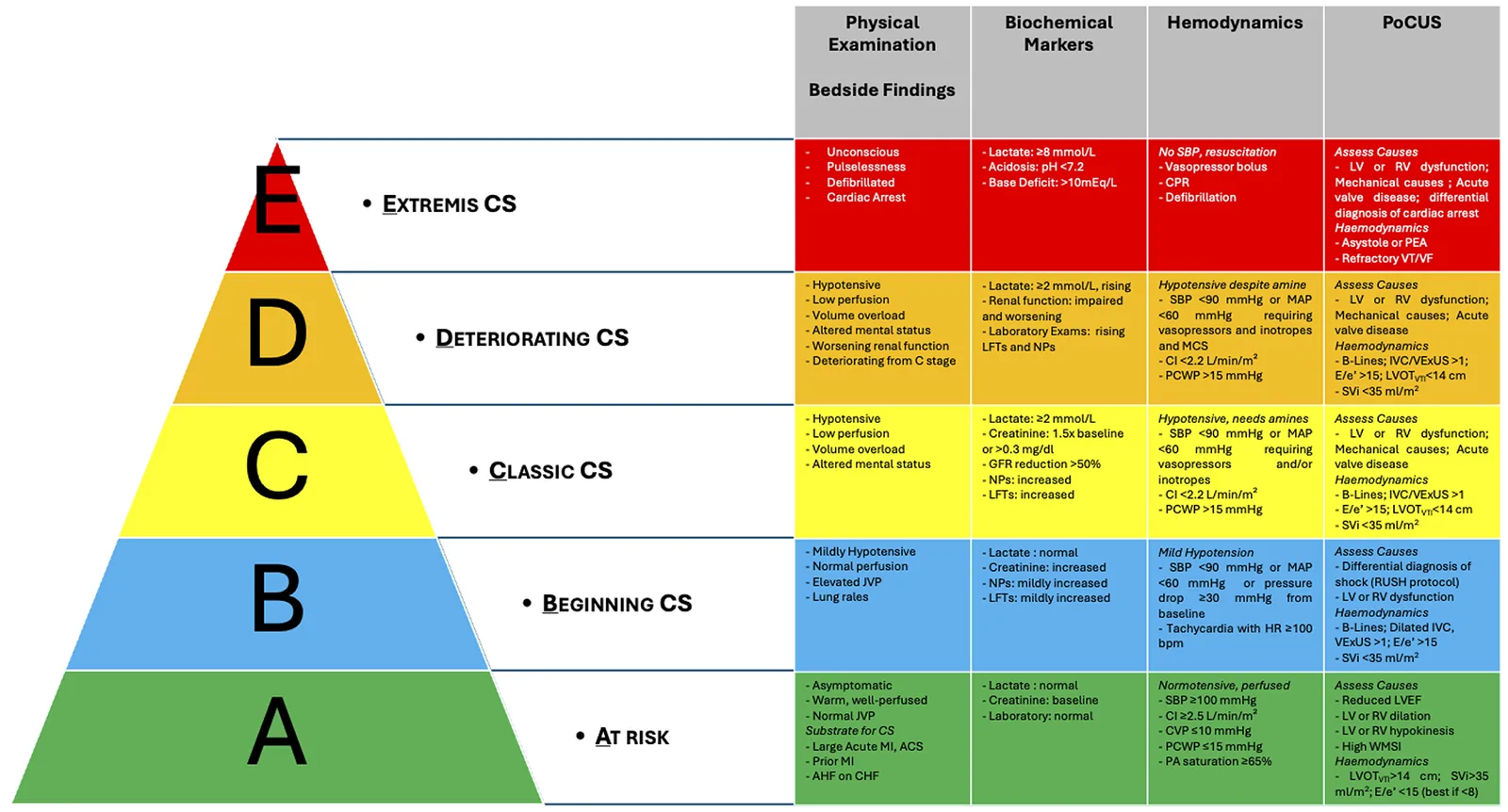
**Integration of PoCUS in the SCAI classification**. Legend: ACS, acute coronary syndrome; AHF, acute heart failure; CHF, chronic heart failure; CI, cardiac index; CS, cardiogenic shock; CVP, central venous pressure; HR, heart rate; IVC, inferior vena cava; JVP, jugular venous pressure; GFR, glomerular filtration rate; LFTs, liver function tests; LV, left ventricle; LVEF, left ventricular ejection fraction; LVOT_VTI_, velocity-time integral at the left ventricle outflow tract; MAP, mean arterial pressure; MI, myocardial infarction; NPs, natriuretic peptides; PEA, pulseless electrical activity; PCWP, pulmonary capillary wedge pressure; RV, right ventricle; SBP, systolic blood pressure; SVi, stroke volume indexed by BSA; VExUS, venous excess ultrasound; VT/VF, ventricular tachycardia/ventricular fibrillation; WMSI, wall motion score index.

Rather than defining CS stages in isolation, PoCUS findings should be interpreted as dynamic markers that evolve progressively across the SCAI spectrum, supporting, but not replacing, clinical, biochemical, and hemodynamic criteria, as shown in Fig. 1.

The same PoCUS parameters recur throughout all SCAI stages; what distinguishes one stage from another is the magnitude, persistence, and temporal progression of abnormalities, as well as their response to initial therapy. Moreover, PoCUS is very important at every SCAI stage to perform a differential diagnosis, especially to identify causes other than CS of hemodynamic instabilization: especially among elderly patients, the presence of a substrate, particularly a chronic or subacute cardiac disease, does not necessarily imply an evolution in CS. HS and DS are the most common causes of hemodynamic instability in this population, and they must always be ruled out with clinical, laboratory, and PoCUS assessment before considering CS, which is a relatively uncommon cause of hemodynamic derangement compared with other causes of undifferentiated shock [178]. Particularly, both HS and DS represent the leading causes of ED admission for undifferentiated shock, with HS often complicating ACS, AMI or post-MI course, representing a risk factor for adverse prognosis [178,179].

In SCAI stage A (at risk), PoCUS primarily serves to identify a structural or functional substrate predisposing the individual to CS, such as reduced biventricular systolic function, ventricular dilation, prior regional wall motion abnormalities, or chronic valvular disease. In subjects with ACS, AMI, previous MI and CHF, PoCUS is helpful in assessing global LV function with LVEF, global RV function with TAPSE or FAC, RV and LV telediastolic diameter, the thickness and kinetics of each LV segment and the synthesis of the function of all LV segments with the wall motion score index (WMSI). Hemodynamic parameters are usually preserved: LUS shows absent or sparse B-lines, IVC diameter is typically <21 mm with preserved collapsibility, VExUS score is low, E/e′ ratio is within normal limits, SV, CO and CI are generally normal.

In SCAI stage B (beginning shock), PoCUS findings often reveal early hemodynamic imbalance, even in the absence of overt hypoperfusion. Mild pulmonary congestion may appear on LUS, IVC diameter may increase over 21 mm with reduced collapsibility, and VExUS score may increase, indicating incipient systemic congestion. Left ventricular filling pressures (E/e′) can be moderately increased, while SV and CI are frequently preserved or only mildly reduced. Comparison with baseline or prior examinations is particularly valuable at this stage to detect clinically meaningful changes.

SCAI stage C (classic cardiogenic shock) is characterised by overt PoCUS evidence of hemodynamic compromise. Pulmonary congestion becomes more pronounced, often with diffuse B-lines and pleural effusions. IVC dilation and elevated VExUS scores reflect significant venous congestion. Biventricular systolic dysfunction is commonly evident, mechanical complications or acute valvular lesions may be detected, and forward flow parameters deteriorate: LVOT velocity–time integral (VTI) is typically reduced (<14 cm), SV and CI fall below normal thresholds (CI often <2.2 L/min/m^2^), consistent with established hypoperfusion.

In SCAI stage D (deteriorating shock), PoCUS is primarily used for serial reassessment, documenting worsening congestion, further reduction in SV and CI, or failure of hemodynamic parameters to improve despite escalating pharmacological or mechanical support. At this stage, ultrasound supports therapeutic optimisation (fluids, inotropes, vasopressors) and may prompt early consideration of mechanical circulatory support or reassessment for alternative or additional diagnoses.

In SCAI stage E (extremis), PoCUS is integrated into resuscitation-focused protocols to identify potentially reversible causes of circulatory collapse, recognise true pulseless electrical activity (PEA), and guide ongoing resuscitative efforts while minimising interruptions to cardiopulmonary resuscitation. At this stage, the Focused Echocardiographic Evaluation in Resuscitation (FEER) protocol provides a structured framework for integrating PoCUS into cardiac arrest and peri-arrest management, aiming to improve diagnostic accuracy while preserving high-quality CPR [180,181,182]. A principal function of FEER is the identification of reversible aetiologies of cardiac arrest, with particular emphasis on cardiac tamponade, high-risk pulmonary embolism, and severe hypovolemia [180,181,182,183]. FEER also helps distinguish true from false PEA, specifically between PEA and pseudo-PEA. The presence of cardiac activity on ultrasound has been associated with higher rates of return of spontaneous circulation (ROSC) and survival than cardiac standstill [184,185,186]. This distinction supports escalation of resuscitative efforts and individualised hemodynamic management. In addition, FEER contributes to real-time resuscitation guidance, informing decisions regarding volume resuscitation, vasopressor use, airway and ventilation strategies, and procedural interventions [180,181]. Serial assessments during rhythm checks may also aid prognostication, as persistent cardiac standstill on repeated examinations is associated with poor outcomes; however, ultrasound findings should not be used in isolation to terminate resuscitation [184,185,187]. A core principle of the FEER protocol is the minimisation of CPR interruptions: image acquisition is restricted to brief rhythm or pulse checks, typically limited to less than 10 seconds, with probe positioning performed during ongoing compressions [180,181,182]. This disciplined approach ensures that PoCUS functions as an adjunct rather than a detractor from evidence-based resuscitation.

## 4. PoCUS in Cardiogenic Shock: From Bedside Phenotyping to Therapeutic Optimisation

CS represents a challenging condition in emergency medicine, owing to its heterogeneous aetiology, dynamic pathophysiology, and persistently high mortality despite advances in pharmacological treatments, reperfusion strategies and MCS. Although CS accounts for a minority of shock presentations, its clinical course is often rapidly progressive, evolving from compensated hemodynamic instability to multiorgan failure and death. In this context, early recognition, accurate phenotyping, and timely escalation of therapy are the principal determinants of outcome.

Our review highlights the role of PoCUS as a first-line diagnostic and monitoring method in patients with suspected CS, especially in the ED. Traditional clinical examination and laboratory markers alone frequently lack sufficient sensitivity and specificity to distinguish CS from other shock phenotypes, particularly in the early or normotensive stages. Conversely, multiorgan PoCUS protocols—selected according to the clinical scenario (eFAST in trauma, RUSH or SHoC in undifferentiated shock, FEER in cardiac arrest)—allow a fast bedside identification of ventricular dysfunction, acute mechanical complications, volume status, pulmonary congestion, and an accurate assessment of alternative causes of shock, improving diagnostic accuracy and clinician confidence [32]. Moreover, a PoCUS structured approach is a key rule-out tool in the early evaluation of the undifferentiated patient with shock, enabling rapid bedside exclusion of alternative etiologies and narrowing the differential diagnosis at presentation. Structured approaches demonstrate high diagnostic accuracy for shock phenotyping and support early management decisions [32]. Notably, this rule-out function may be among the most useful PoCUS applications in time-sensitive settings, although its diagnostic contribution does not necessarily translate into improved patient-centred outcomes without integration into timely therapeutic pathways. A key contribution of PoCUS in CS lies in its ability to help differentiate ischemic CS (ACS-CS, AMI-CS, or post-MI-CS) from AHF-CS, which often exhibit distinct clinical and echocardiographic patterns. Focal wall-motion abnormalities and mechanical complications are typical of post-myocardial infarction CS, whereas diffuse ventricular dysfunction characterises myocarditis, Takotsubo syndrome, or ADHF. This distinction is clinically relevant, as it influences early therapeutic decisions, including revascularisation strategies, inotropic support, and mechanical circulatory support selection [46,47].

Beyond diagnosis, PoCUS plays a pivotal role in hemodynamic profiling and severity stratification, particularly when integrated with the SCAI classification. This framework provides a shared language for staging CS severity, but its hemodynamic component relies on timely and accurate assessment of cardiac function and congestion. PoCUS directly informs this pillar by enabling rapid evaluation of biventricular performance, filling pressures, venous congestion, and systemic perfusion. In this regard, PoCUS supports earlier identification of patients transitioning from “at risk” or “beginning” shock (SCAI A–B) to overt hypoperfusion (SCAI C–D), for which prompt escalation of therapy may be lifesaving [2]. Moreover, PoCUS is evolving into a key tool for real-time therapeutic guidance and hemodynamic optimisation in CS. By enabling bedside hemodynamic phenotyping, PoCUS directly informs treatment selection. A small, collapsible IVC or a positive passive leg-raising test suggests fluid responsiveness and should prompt volume expansion, whereas signs of elevated filling pressures and systemic congestion (dilated, non-collapsible IVC, pulmonary B-lines, or a high VExUS grade) discourage fluid administration and instead favour diuretics or vasoactive support. In addition, serial PoCUS assessment enables dynamic treatment titration, allowing clinicians to monitor changes in stroke volume surrogates, ventricular performance, pulmonary and systemic congestion in response to fluids, vasopressors, or inotropes, thereby minimising the risk of under- and over-resuscitation. Importantly, contemporary management strategies increasingly emphasise fluid tolerance and avoidance of venous congestion rather than fluid responsiveness alone. In this context, PoCUS-derived markers such as IVC dynamics, LUS B-lines, and VExUS patterns provide actionable information to optimise preload while minimising iatrogenic harm [12,83]. PoCUS also facilitates the identification of specific therapeutic targets, including mechanical complications, pericardial tamponade requiring urgent drainage, or RV failure requiring tailored preload and afterload optimisation. Furthermore, when integrated with clinical and biochemical parameters, PoCUS supports the early recognition of treatment failure and timely escalation to advanced therapies, including MCS, in accordance with SCAI stage-based management strategies.

### Limitations

Despite the growing adoption of advanced bedside diagnostic tools in cardiogenic shock, a critical distinction must be made between diagnostic accuracy and clinical effectiveness. Contemporary studies demonstrated that PoCUS achieves high diagnostic performance in identifying CS [32]. However, the certainty of this evidence is limited by heterogeneity, operator dependency, and predominance of observational design studies. Moreover, improvements in diagnostic accuracy have not consistently translated into meaningful benefits in patient-centred outcomes: a recent systematic review incorporating randomised data found no significant reduction in in-hospital mortality associated with the use of PoCUS in suspected cardiogenic shock (RR: 0.99; 95% Confidence Interval: 0.64–1.51) [188]. Similarly, contemporary scoping reviews report no significant effects on mortality, ICU length of stay, or the need for organ support, despite signals of improved early hemodynamic characterisation [189]. This observed effect likely reflects the complex, time-sensitive nature of CS, in which outcomes are driven primarily by the timely implementation of effective therapies (such as revascularisation, electrical cardioversion, mechanical circulatory support, and pharmacologic strategies) rather than by diagnostic refinement alone. Moreover, despite its strengths, PoCUS remains operator-dependent and should not be interpreted in isolation. Image quality limitations, inter-operator variability, and the absence of universally standardised acquisition protocols underline the importance of structured training and integration within a multidisciplinary diagnostic framework. The gap between diagnostic performance and clinical benefit underscores the need for further research into integrated care pathways that link the potential of an early diagnosis and the capability of a patient’s physiology assessment in the ED to guide evidence-based therapeutic actions.

## 5. Conclusion

CS is a complex, time-sensitive syndrome in which early diagnosis, accurate phenotyping, and continuous hemodynamic reassessment are essential in improving outcomes. This narrative review underscores the role of PoCUS in the contemporary management of CS, enabling fast differential diagnosis, identification of reversible causes, and real-time monitoring of cardiac function and congestion. When integrated with standardised protocols and the SCAI classification, PoCUS enhances early recognition of CS, allows adequate stratification of its severity, and supports personalised, physiology-guided therapy, thereby facilitating timely escalation to advanced circulatory support, when indicated. Its repeatability, bedside availability, and non-invasive nature make PoCUS uniquely suited for dynamic decision-making in emergency and critical care settings. Future research should focus on prospective studies evaluating PoCUS-guided treatment algorithms and their specific impact on CS clinical outcomes, as well as on the standardisation of training and competency assessment. Until such data are available, PoCUS should be regarded as an indispensable adjunct to clinical judgement, bridging the gap between bedside examination and advanced hemodynamic monitoring in patients with CS.
